# Landscape-level bird loss increases the prevalence of honeydew-producing insects and non-native ants

**DOI:** 10.1007/s00442-018-4273-5

**Published:** 2018-10-26

**Authors:** Micah G. Freedman, Ross H. Miller, Haldre S. Rogers

**Affiliations:** 1 Center for Population Biology, University of California, Davis, Davis, CA 95616 USA; 2 Department of Evolution and Ecology, University of California, Davis, Davis, CA 95616 USA; 3 College of Natural and Applied Science, University of Guam, Mangilao, GU 96913 USA; 4 Department of Ecology, Evolution, and Organismal Biology, Iowa State University, Ames, IA 50014 USA

**Keywords:** Bird loss, Ants, Honeydew-producing insects, Islands, Trophic cascade

## Abstract

Bird exclusion experiments consistently show that birds exhibit strong top-down control of arthropods, including ants and the honeydew-producing insects (HPIs) that they tend. However, it remains unclear whether the results of these small-scale bird exclosure experiments can be extrapolated to larger spatial scales. In this study, we use a natural bird removal experiment to compare the prevalence of ants and HPIs between Guam, an island whose bird community has been extirpated since the 1980s due to the introduction of the brown tree snake, and two nearby islands (Rota and Saipan) that have more intact bird assemblages. Consistent with smaller-scale bird exclosure experiments, we show that (1) forest trees from Guam are significantly more likely to host HPIs than trees from Saipan and (2) ants are nearly four times as abundant on Guam than on both Saipan and Rota. The prevalence of HPIs varied slightly based on tree species identity, although these effects were not as strong as island-level effects associated with bird loss. Ant community composition differed between Guam and the other two islands. These results corroborate past observational studies showing increased spider densities on Guam and suggest that trophic changes associated with landscape-level bird extirpation may also involve alterations in the abundance of ants and HPIs. This study also provides a clear example of the strong indirect effects that invasive species can have on natural food webs.

**Electronic supplementary material:**

The online version of this article (10.1007/s00442-018-4273-5) contains supplementary material, which is available to authorized users.

## Introduction

Removal of apex predators from an ecosystem can have cascading effects on organisms occupying lower trophic positions. Experimental evidence from numerous systems has shown that the loss of top predators—including wolves (Ripple et al. 2001), sea otters (Estes and Duggins 1995), and starfish (Paine 1980)—can profoundly impact the abundance of remaining community members and their trophic dynamics. This loss of top predators, a process sometimes referred to as trophic downgrading, is thought to be one of humankind’s most pervasive influences on natural communities (Estes et al. 2011).

One guild of predators whose experimental removal provides extensive support for the idea of trophic cascades is vertebrate insectivores. Studies involving exclusion of lizards (Spiller and Schoener 1990, 1994), bats (Kalka et al. 2008; Williams-Guillén et al. 2008), and birds (Mooney 2006; Van Bael et al. 2008; Philpott et al. 2009; Bridgeland et al. 2010) all demonstrate the strong impacts these insectivores can have on their arthropod prey. In a meta-analysis of 113 studies that excluded vertebrate insectivores, Mooney et al. (2010) showed that these predators significantly reduce the abundance of arthropods and thereby reduce damage to plants. However, these experiments are conducted at small spatial scales, often involving exclusion of insectivores from individual plants or single branches. In contrast, many fewer studies have addressed the landscape-level effects of vertebrate insectivore removal (but see Spiller and Schoener 1997; Rogers et al. 2012), in part because of logistical and/or ethical constraints associated with this kind of manipulation.

Experimental removal of predators is expected to affect arthropod groups differently depending on their trophic position (Mooney et al. 2010). Among the arthropods that appear to benefit most strongly from vertebrate insectivore removal are honeydew-producing insects (hereby HPIs) and the ants that feed on their excreted honeydew (Mooney 2006, 2007). Mooney (2006) showed that bird exclusion from Ponderosa pine (*Pinus ponderosa*) increased the abundance of aphids even in the absence of tending ants, and that the positive effects of bird exclusion on aphid abundance become especially pronounced when tending ants are allowed to associate with aphids. Similarly, Mooney (2007) showed that birds and ants interacted significantly to determine the abundance of tended aphid species, with ants only increasing the abundance of these aphids significantly when birds were excluded. Maas et al. (2013) demonstrated that exclusion of birds and bats from commercial cacao plantations significantly increased the abundance of both aphids and their tending ants.

Although these studies provide experimental evidence for the role of vertebrate insectivores in mediating ant and HPI abundance, they involve single species of host plants distributed over relatively small spatial scales and small-scale insectivore exclusion treatments. Thus, it remains unclear how loss of insectivores at the landscape level might affect ant–HPI associations. Meta-analyses have shown that the size of experimental insectivore exclosures does not correlate with estimates of top-down trophic control by birds (Mooney et al. 2010; Mäntylä et al. 2011), although these studies typically involve manipulations at the scale of less than 10 m^2^, and never exceeding 750 m^2^. Because ant–HPI associations can be keystone interactions with dramatic impacts for their constituent communities (O’Dowd et al. 2003; Kaplan and Eubanks 2005), it is important to understand how landscape-level loss of vertebrate insectivores may affect ant–HPI associations.

Bird exclusion is expected to increase the abundance of both ants and the HPIs that they tend, although the effects of bird exclusion may in part be mediated through bottom-up processes related to host plant identity. Many studies have investigated how vertebrate exclusion affects ants and HPIs on single host plant species (see above references), and others have examined how plant variation—both intraspecific variation in traits (Johnson 2008; Moreira and Mooney 2013; Züst and Agrawal 2017) and species diversity of plants (Staab et al. 2015)—affects ant-HPI interactions. However, it remains unclear whether vertebrate exclusion leads to uniformly increased abundance of ants and HPIs across plant species, or whether some plant species become more susceptible to ant–HPI associations upon vertebrate exclusion.

In addition to changing the numerical abundance of HPIs and associated ants, loss of vertebrate insectivores could also influence the community composition of ants based on their trophic position. Ant species vary in their dietary flexibility and their propensity to form associations with HPIs (Carroll and Janzen 1973; Helms and Vinson 2002; Tillberg et al. 2007). In particular, a number of highly invasive ant species are thought to be especially adept at forming facultative mutualisms with HPIs (Lach 2003; Savage et al. 2009, 2011). Thus, the exclusion of vertebrate insectivores is expected to directly benefit both ants and HPIs through reduced predation, as well as indirectly favoring ant species that are most capable of capitalizing on the abundant carbohydrate resources provided by HPIs.

**Fig. 1:**
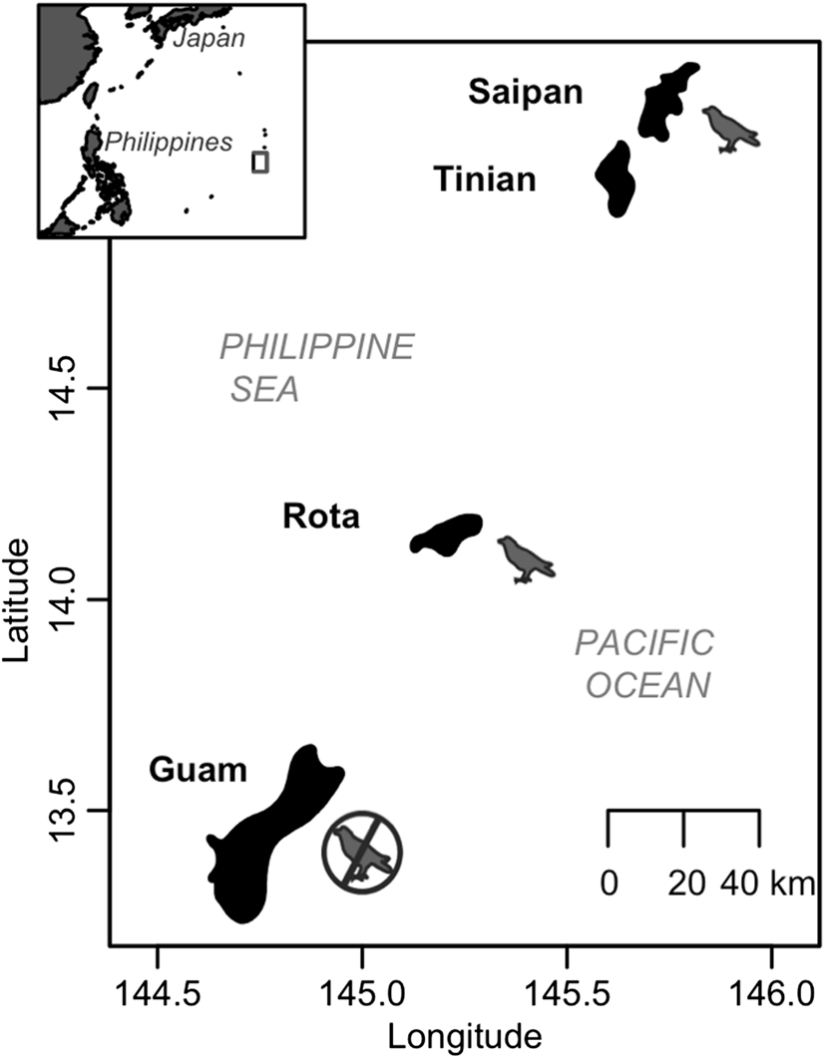
Map of the Mariana Islands. Guam has no functional insectivorous bird predation. Rota and Saipan both have mostly intact communities of insectivorous birds, although densities are likely higher on Saipan than on Rota

In this study, we use the Mariana Islands as a natural experiment to investigate the landscape-level impacts of vertebrate insectivore loss for ant–HPI associations. We compare Guam, an island whose bird assemblage has been functionally extirpated since the 1980s due to the introduction of the brown tree snake (Savidge 1987) to the nearby islands of Rota and Saipan, which both have bird assemblages that are largely intact (Fig. 1). We sample ants and HPIs from trees in native limestone karst forest to address the following questions: (1) Are ants and HPIs more prevalent when vertebrate insectivores are reduced? (2) Does ant and HPI prevalence vary among tree species? (3) Are declines in vertebrate insectivores associated with changes in ant community composition? For question 1, we expect to find increases in both ant and HPI abundance on Guam, consistent with smaller-scale vertebrate exclusion experiments. For the second question, we expect that certain tree species will be especially susceptible to colonization by HPIs and their attending ants, but we do not expect vertebrate exclusion to alter abundance of ants and HPIs in a species-specific manner. For the third question, we expect that the loss of birds on Guam will favor ant species adept at monopolizing Hemipteran honeydew resources and lead to a different community composition of ants than on Saipan and Rota.

## Methods

### Study system

We sampled ants and HPIs from three islands—Guam (544 km^2^), Rota (85 km^2^), and Saipan (180 km^2^)—in the Mariana Islands chain in the western Pacific (Fig. 1). Sampling sites were all located in relatively undisturbed karst limestone forest that contained predominantly native forest trees. The Mariana Islands have 57 recorded ant species, almost all of which are thought to be recent human-assisted introductions (Clouse 2007). All of the ant species recorded in this study are thought to be human introductions, with the possible exceptions of *Odontomachus simillimus* and *Camponotus* sp. (Table S1). The identities of most HPIs recorded in this study are not known. However, most are likely to also be recent human-assisted introductions; for example, Miller et al. (2014) indicate that all 35 species of aphids recorded in Micronesia are introduced, and similar patterns have been documented for the aphid assemblages of Hawaii (Messing et al. 2007) and Palau (Idechiil et al. 2007).

The introduction of the brown tree snake (*Boiga irregularis*) to Guam in the 1940s led to the almost complete extirpation of the island’s avifauna by the 1980s (Savidge 1987). The only remaining insectivorous forest bird species are the omnivorous Micronesian starling (*Aplonis opaca*), which occurs primarily in developed areas in the northern tip of the island, and the Mariana swiftlet (*Aerodramus bartschi*), restricted to three caves in southern Guam far from our study areas.

The nearby islands of Rota and Saipan have intact bird assemblages when compared to Guam, including forest-dwelling insectivores such as Rufous fantails (*Rhipidura rufifrons*) and white-eyes (*Cleptornis marchei*, *Zosterops conspicillatus*, *Z. rotensis*) that likely encounter arboreal ants and HPIs. Recently estimated densities of Rufous fantails are slightly higher on Rota (651 ± 16 individuals/km^2^) compared to Saipan (469 ± 63 individuals/km^2^), although Saipan has much higher densities of bridled white-eyes (*Z. conspicillatus*) (4713 ± 387 individuals/km^2^) and golden white-eyes (*C. marchei*) (711 ± 112 individuals/km^2^) compared to densities of Rota white-eyes (*Z. rotensis*) (450 ± 14 individuals/km^2^) (Camp et al. 2009, 2015); Rota white-eyes have not been recorded at our study sites. Finally, Saipan also has insectivorous Mariana swiftlets that are locally extirpated from Rota. Together, this indicates that the overall densities of insectivorous birds are likely higher on Saipan than on Rota, but both have significantly more native birds than Guam.

In addition to the loss of the island’s avifauna, Guam also likely has many fewer insectivorous lizards than Saipan and Rota. Rodda and Fritts (1992) sampled skinks and geckos from Guam and three nearby islands in the Marianas and showed that the biomass of insectivorous geckos is significantly lower on Guam. Although biomass of ground-dwelling skinks was still comparable between islands (Rodda and Fritts 1992), these surveys suggest that predation of arthropods by arboreal lizards is also likely to be lower in Guam compared to Rota and Saipan.

### Sampling protocol

We selected 13 tree species that commonly occur in the native limestone forest communities of the Mariana Islands for sampling ant and HPI communities (Table 1). Focal trees were located in three 1 ha (100 m × 100 m) forest plots on Rota, three plots on Saipan, and five plots on Guam. Within each tree species, we randomly selected individual trees from a pre-existing database of approximately 20,000 tagged adult trees across these 11 forest plots. Trees were not chosen if their canopies overlapped with other sampled trees to minimize spatial pseudo-replication that might result from shared ant or HPI communities. Each tree was sampled once during a 3-week period in January and February of 2014, which corresponds to the dry season for the Mariana Islands.

**Table 1:** Number of trees sampled from each island

Tree species	Guam	Rota	Saipan	Total
Aglaia mariannensis* (Meliaceae)	11	4	8	23
Cynometra ramiflora* (Fabaceae)	8	1	7	16
*Eugenia reinwardtiana* and *E. palumbis* (Myrtaceae)*	9	3	6	18
*Ficus* spp.* (Moraceae)	3	1	3	7
*Leucaena leucocephala* (Fabaceae)	0	3	6	9
Macaranga thompsonii* (Euphorbiaceae)	17	3	0	20
Meiogyne mariannae* (Annonaceae)	12	3	7	22
*Morinda citrifolia* (Rubiaceae)	9	2	6	17
Ochrosia oppositifolia* (Apocynaceae)	11	4	5	19
Ochrosia mariannensis* (Apocynaceae)	11	4	5	20
Premna serratifolia* (Lamiaceae)	7	3	6	16
Psychotria mariana* (Rubiaceae)	6	4	6	16
*Triphasia trifolia* (Rutaceae)	6	3	0	9
Cumulative total	106	35	67	208

Species with an asterisk are considered to be native to the Mariana Islands. *Ficus prolixa* and *F. tinctoria* are reported here but were excluded from analyses (see “Methods”)

Once a pre-selected tree was located, a branch was chosen by using a random number generator to give a value between 0 and 360; this value was then treated as a compass bearing and used to walk to the edge of the tree canopy. A small (< 2 cm diameter) branch was carefully clipped from the canopy using a 4.25 m pole saw and allowed to drop to the forest floor; while this method of collection likely led to some ants and HPIs falling off and escaping detection, we were consistent in our sampling across tree species and islands, so there should not be systematic bias in our estimates. This clipped branch was promptly examined for the presence and abundance of ants and any herbivores, and the number of leaves on the sampled branch was also recorded. Any ant tending behavior (i.e., ants in close proximity to HPIs in proctodeal orientation) was recorded. Counts of ants ascending or descending from the trunks of sampled trees were also made by choosing a fixed reference line on the trunk of the tree and recording the number of ants that crossed this line over a 1 min observation period; this measurement was used as an estimate of ant abundance for each sampled tree. Ants were collected and stored in ethanol vials for later identification.

### Species identification

Ant specimens stored in ethanol were identified using the key provided in Clouse (2007). In total, 12 ant species were positively identified from canopy branches (Table S1). Because we use the nomenclature presented in Clouse (2007), we treat the species of *Technomyrmex* encountered on Guam as *T. albipes*. However, we note that other published reports suggest that many records of *T. albipes* are actually likely to be *T. difficilis* (Bolton 2007). We recorded all putatively herbivorous arthropods present on the leaves of sampled branches and determined whether they were HPIs based on presence of ant tending, visible honeydew excretions, and published reports of ant associations. The primary HPIs present were aphids (Aphididae), soft scales (Coccidae), mealybugs (Pseudococcidae), leafhoppers (Membracidae), and whiteflies (Aleyrodidae) (Figure S2). We recorded 16 distinct HPI morphospecies, although some of these were likely single species in different developmental stages. Fourteen of these HPI morphospecies were observed to be tended by ants. Interactions between HPIs and host plants are summarized in Figure S2.

### Data analysis

Our first two questions sought to address (1) whether ant and HPI prevalence differs between islands and (2) whether these effects are related to tree species identity. To address these questions, we used generalized linear mixed models implemented in the lme4 package (Bates et al. 2015) in R version 3.4.1 (R Core Development Team 2017).

For all of the models tested, we included island (categorical variable), tree species (categorical variable), and the presence of ants (for models with HPIs as a response) or HPIs (for models with ants as a response). Sampling site was included as a random intercept to account for spatial covariance between samples within islands. Leaf number was included as a covariate to account for differences in detection probabilities association with the size of sampled branches. We first fit full models with all two- and three-way interactions between island, tree species, and HPI/ant presence, and then used Akaike information criteria scores with small sample size correction (AICc) to determine whether model fits were improved without these interaction terms. We considered models with ΔAICc of 2.0 or greater as having better model fit. Model fits were improved by excluding interaction terms for all analyses (Table S2); thus, subsequent analyses and reported figures were based on models that included all possible covariates but no interactions between them. Pairwise comparisons within factor levels were made using the glht function implemented in the multcomp package (Hothorn et al. 2008) (Table 2). Two tree species (*Ficus prolixa* and *F. tinctoria*) were omitted from data analysis because of their combined low sample size (*n* = 3 on Guam, *n* = 1 on Rota, *n* = 3 on Saipan) and inflated standard error estimates; omission of *Ficus* spp. did not affect the values of other estimated parameters.

**Table 2:** Log odds and associated confidence intervals for comparisons between factor levels

Pairwise comparison	Estimate	Lower CI	Upper CI
Model 1—response: HPI presence			
Guam vs. Rota	0.47	− 1.47	2.37
**Guam vs. Saipan**	**1.85**	**0.02**	**3.69**
Rota vs. Saipan	1.39	− 0.85	3.61
**Ants present vs. ants absent**	**1.77**	**0.71**	**2.82**
Model 2—response: ant presence			
Guam vs. Rota	1.44	-0.37	3.24
Guam vs. Saipan	0.67	− 0.96	2.30
Rota vs. Saipan	− 0.77	− 2.71	1.18
**HPIs present vs. HPIs absent**	**1.76**	**0.72**	**2.80**
Model 3—response: ant abundance			
**Guam vs. Rota**	**1.38**	**0.27**	**2.48**
**Guam vs. Saipan**	**1.13**	**0.13**	**2.13**
Rota vs. Saipan	− 0.24	− 1.45	0.95
HPIs present vs. HPIs absent	0.34	− 0.24	0.91

Comparisons whose 95% CIs do not overlap with 0 are considered to be significant and are highlighted in bold. Pairwise comparisons between tree species are not reported because only one comparison (*Macaranga* vs. *Cynometra*) was significantly different from 0 for model 1, and none were significant for models 2 and 3

For the first analysis, HPI presence/absence (1/0) was used as the response variable. HPIs were present on 20.5% (7/34) of branches from Rota, 9.3% (6/64) of sampled branches on Saipan, and 44.7% (46/103) of sampled branches on Guam. We did not use HPI abundance as our response variable because of the pronounced size differences between groups of HPIs (e.g., a single membracid is many times larger than a single aphid) and the ability for many HPIs to reproduce parthenogenetically. For the second analysis, ant presence/absence (1/0) on sampled branches was used as a response variable. Both models were fit with a binomial error structure and logit link function to reflect the binary nature of our response variable.

To assess differences in ant abundance on the trunks of sampled trees, we used counts of individual ants as a response variable. This model was fit with a negative binomial error structure using glmer.nb from the MASS package (Venables and Ripley 2002).

Our third question asked whether ant community composition differed between islands. To determine this, we used non-metric multidimensional scaling (NMDS) in the vegan package of R (Oksanen et al. 2017) and built a community matrix of ant abundances using counts of individual ants found on branches and trunks. Based on the results of a scree plot, which shows stress as a function of dimensionality, we used *k* = 6 dimensions and simulated over 2500 possible random starts to generate a matrix of Bray–Curtis dissimilarity values, which take into account information on both the presence–absence and relative abundance of community members. Trees with no ants were excluded from this analysis, leaving us with 113 total sampled branches. We used analysis of similarity (ANOSIM) to determine the impact of island, site, and tree species for determining ant community composition.

We also performed a similar analysis using the community of herbivorous arthropods found on each branch. However, due to the relatively small number of observations containing HPIs (*n* = 59 total), our distance matrix was too sparse for multivariate analysis of HPI communities, and so the results presented in Figure S3 reflect non-HPI herbivores as well. To visualize pairwise interactions between HPIs and tree species, we created a bipartite network in the “bipartite” package (Dormann et al. 2008).

We used the manyglm function with PIT-trap resampling and a negative binomial distribution, implemented in the mvabund package (Wang et al. 2012), to determine the relative importance of individual ant species in shaping overall community similarity. This approach fits a generalized linear model using a common set of predictor variables to the abundance of each individual species in the community matrix, and then assesses the change in likelihood that accompanies the removal of a single species from the community matrix.

## Results

**Fig. 2:**
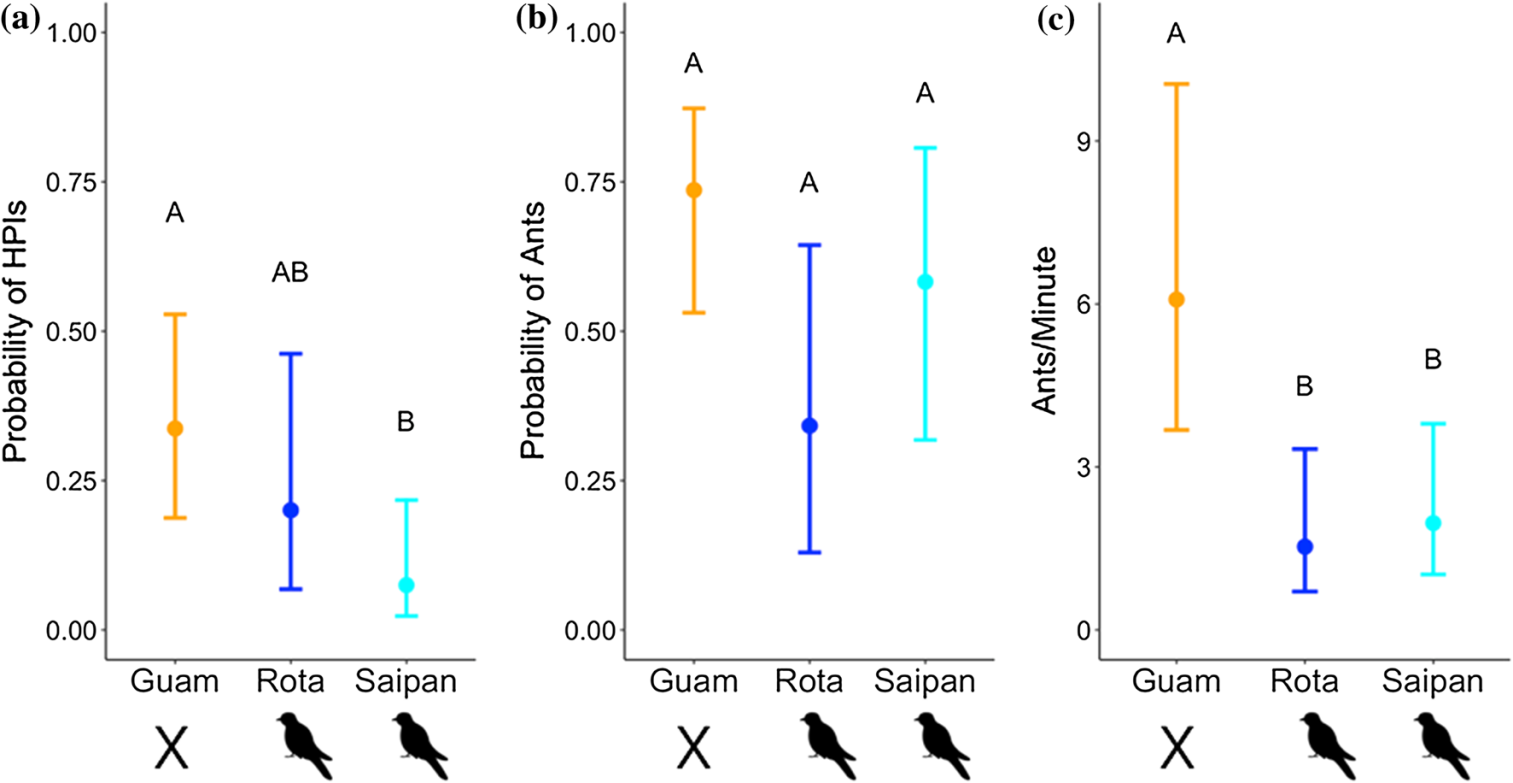
Model results showing **a** probability of detecting HPIs on sampled branches, **b** probability of detecting ants on sampled branches, **c** abundance of ants on trunks of sampled trees. Error bars represent 95% confidence intervals, and letters correspond to group-level differences after correction for multiple comparisons

The presence of ants and HPIs was strongly positively correlated, and HPI presence also increased ant abundance on tree trunks (Table 1). On branches that had HPIs present, ants were also present in 84.7% (50/59) of observations. In contrast, ants were only present on 47.6% (71/149) of sampled branches lacking HPIs. Guam had a significantly higher proportion of trees with HPIs present than Saipan (Table 1, Fig. 2a); however, the probability of encountering HPIs on Guam was not significantly different than on Rota (Table 1, Fig. 2a). *Macaranga* was significantly more likely to host HPIs than *Cynometra*, although this was the only significant pairwise species-level difference in HPI presence (Figure S1). We recorded 24 instances of active ant tending of HPIs, with 21 of these observations coming from Guam. Among the 21 trophobioses observed on Guam, 17 involved the ant *Technomyrmex albipes*.

In contrast to HPI presence, ant presence was not significantly different between Guam and the other two islands (Table 2, Fig. 2b). Likewise, ant presence between Rota and Saipan was not significantly different (Table 2, Fig. 2b), and no tree species were significantly different from one another in ant presence (Figure S1).

**Fig. 3:**
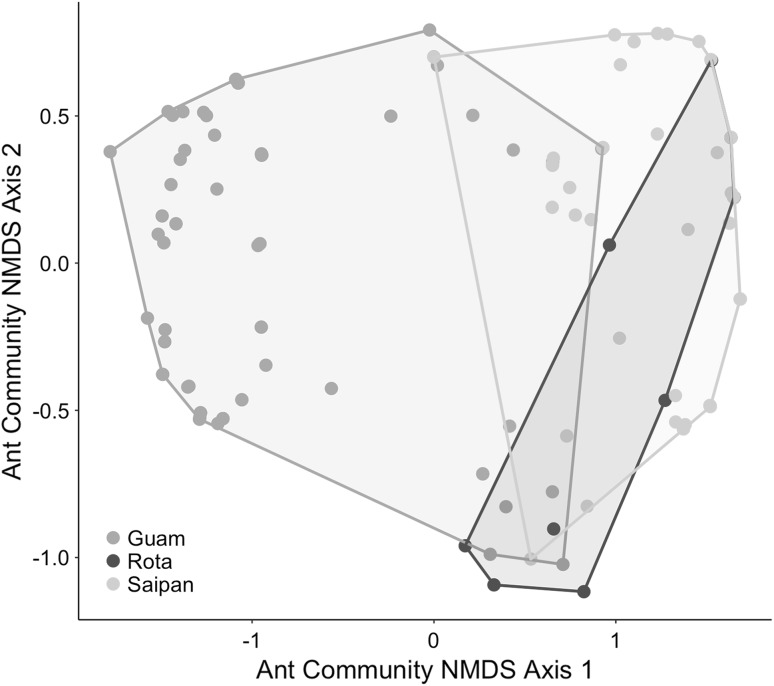
NMDS plot showing ant community composition across islands. Plot is based on six dimensions and Bray–Curtis distances, with a stress value of approximately 0.08. Each point corresponds to the ant community encountered in a single tree. Outlines encompass all of the points for each island. Ant communities between islands are significantly different (ANOSIM *R* = 0.544, *p* < 0.001)

Ants were significantly more abundant on trunks of sampled trees on Guam than on Rota and on Saipan (Table 2, Fig. 2c). Saipan and Rota were not significantly different from one another (Table 2, Fig. 2c). None of the pairwise differences among tree species in ant abundance were significant (Fig. 3).

Ant communities between islands were significantly different from one another (Fig. 3), as confirmed by ANOSIM results (*R* = 0.544, *p* < 0.001). Sites within islands also had significantly different ant assemblages (*R* = 0.492, *p* < 0.001). Tree species identity was a weaker predictor of ant community composition (*R* = 0.025, *p* = 0.108) than island or site. The species that contributed most strongly to ant community composition were *Technomyrmex albipes* (AIC = 515), *Anoplolepis gracilipes* (AIC = 214), and *Tapinoma melanocephalum* (AIC = 199).

## Discussion

Our primary question asked whether the prevalence of ants and HPIs was higher on the bird-free island of Guam. Among the three islands sampled, Guam indeed had the highest abundance of ants and also was significantly more likely to support HPIs than Saipan. This effect takes into account differences in ant–HPI associations between different tree species and is therefore unlikely to be driven by compositional differences in forest trees between islands. This result is in agreement with previous studies that have experimentally excluded birds at small spatial scales and recorded concomitant increases in ants and HPIs (Mooney 2006, 2007, Maas et al. 2013) and a recent meta-analysis showing strong top-down control of sucking insects (Vidal and Murphy 2018). These results are also similar to those found by Rogers et al. (2012), who showed that the abundance of web-building spiders is significantly higher on Guam than nearby islands with intact bird assemblages.

Our findings indicate that the mechanisms driving increases in ants and HPIs in smaller-scale vertebrate exclusion experiments also apply at landscape scales. This supports the findings of meta-analyses showing no effect of exclosure size on top-down trophic control by birds (Mooney et al. 2010; Mäntylä et al. 2011). Likewise, it is consistent with findings of Spiller and Schoener (1997), who showed that island-level absence of lizards produces increases in leaf herbivory similar in magnitude to those observed in manipulative lizard exclosure studies (Spiller and Schoener 1990, 1994).

While we did indeed find increases in ants and HPIs on Guam, it is important to remember that our study includes data from only one bird-free island. Our experimental design thus suffers from pseudo-replication, as all sites without birds are spatially clustered and share attributes beyond just the absence of birds. This shortcoming limits our power of inference and makes it difficult to ascribe causation for the observed increases in ants and HPIs on Guam. For example, increases in HPIs on Guam could be indirect and related to increased densities of web-building spiders—due either to release from top-down control by birds (Rogers et al. 2012) or increased densities of ants (Schuldt and Staab 2015)—that could in turn limit natural enemies of HPIs.

Although Guam had a significantly higher proportion of trees with HPIs than Saipan, the difference in HPI prevalence between Guam and Rota was not statistically significant. One possible reason for the lack of a difference in HPIs between Guam and Rota is the relatively small number of sampled trees from Rota (*n* = 34 total). A previously published meta-analysis of bird exclusion experiments indicates a mean log response ratio of − 0.47 for Hemipterans in the presence of birds (Mooney et al. 2010). Applying this value to our sampling design (*n* = 108 branches from Guam), we would have required a minimum of 54 sampled branches from Rota to detect a significant inter-island difference between Guam and Rota with 80% certainty. Thus, we suggest that the lack of a difference in HPIs between Guam and Rota is mostly an issue of low statistical power. Rota’s intermediate level of HPIs could also reflect the lower abundance of insectivorous birds on Rota compared to Saipan (Camp et al. 2009, 2015), which may contribute to reduced direct predation of HPIs by birds. This possibility is corroborated by the findings of Davis et al. (2008), who showed in a similar island system with invasive ants and HPIs that Christmas Island white-eyes (*Zosterops natalis*) forage extensively on ant-tended scale insects.

We also did not find differences between Guam and Rota or Saipan in ant presence on sampled branches. The lack of a difference between islands likely reflects that ant presence on branches is an imprecise measure that also captures ants involved in exploratory foraging or feeding on floral or extrafloral nectar. By contrast, ant abundance based on trunk counts was nearly identical between Rota and Saipan and nearly four times lower than on Guam, suggesting that this may be a better and more precise measure of ant prevalence. We note here that different ant species may recruit at different levels to the same resource (Human and Gordon 1996), and so the observed differences in abundance that we show could in part be driven by differences in the identity of ants present on each island (see below).

Our second question asked whether tree species differed in their propensity to support ant–HPI associations. We found that one species, *Macaranga thompsonii*, was marginally more susceptible to hosting HPIs than the other 11 species we examined (Figure S1). In spite of the slight preference by HPIs for *Macaranga*, our overall results suggest that there is little in the way of host plant filtering of ant–HPI associations in the Mariana Islands. We found no differences between tree species in the abundance of ants on tree trunks or presence/absence of ants on sampled branches. Instead, the occurrence of ant–HPI associations seems to be highly variable and shared by trees within native forests equally (Figure S1). One possibility for the absence of strong host plant filtering of HPIs in our study is that all recorded HPIs are likely recently-established generalists able to feed on a wide range of hosts. This is in contrast to interactions between plants and HPIs in native forests, which are typically characterized by a high degree of host plant specificity (Staab et al. 2015). Finally, we did not find support for a model that included a tree species × island interaction, suggesting that bird exclusion does not disproportionately promote HPIs on specific host plant species.

Although interactions between ants and HPIs can benefit host plants through deterrence of potentially more damaging herbivores (Styrsky and Eubanks 2007), their impacts on native forest trees of Guam are likely to be negative. Negative impacts on native vegetation due to non-native ant–HPI associations have been documented from numerous island systems, including the Seychelles (Hill et al. 2003), Christmas Island (O’Dowd et al. 2003), and Mauritius (Hansen and Müller 2009). In addition to direct damage through their feeding, HPIs can potentially increase damage by chewing herbivores (Schuldt et al. 2017) and act as vectors for numerous plant diseases (Weintraub and Beanland 2006), and their attending ants can disrupt pollination (Lach 2007; Hansen and Müller 2009) and seed dispersal (Davis et al. 2010; Hansen and Müller 2009). Finally, because nearly all of the ants and HPIs on Guam are recent introductions, limestone forest trees may lack traits that protect against the negative impacts of these ant–HPI associations (e.g., Junker et al. 2011).

Our final question asked whether ant communities differed between islands. Guam indeed had a significantly different ant community structure than Rota and Saipan (Fig. 3). These differences are driven primarily by a single ant species, *T. albipes*, which was the numerically dominant species at 4/5 sites on Guam but entirely absent from all sites on Saipan and Rota. *Technomyrmex albipes* was by far the most prevalent ant species involved in HPI tending, accounting for 17/21 observed trophobioses, suggesting that its prevalence on Guam may be due at least in part to its association with HPIs. Tending by *T. albipes* has been documented as a major reason for the success of the invasive spherical mealybug on Guam (Nechols and Seibert 1985).

There are numerous possible explanations for the differences in ant communities between islands. One possible reason for the numerical dominance of *T. albipes* on Guam is historical contingency (Lester et al. 2009; Fukami 2015), whereby *T. albipes* was an early introduction and reached high enough densities to suppress other ant species that might have otherwise become numerically dominant. Another possibility is that the loss of functional bird predation on Guam, especially coupled with the island’s size and economic activity, has increased the chances of successful establishment by novel ant and HPI species. Yet another possibility is that the loss of bird predators on Guam has favored ant species especially adept at capitalizing on the abundant carbohydrate resources produced by HPIs. For example, in diverse natural plant–HPI–ant communities in the Amazon, Blüthgen et al. (2000) showed that a small number of dominant ant genera monopolized homopteran honeydew resources; a similar dominance hierarchy among ants may explain the extremely high numbers of *T. albipes* on Guam. Whatever the reason for the differences in ant community composition between islands, it is clear that native forests on Guam support an ant community distinct from those found in the forests of nearby islands with insectivorous birds.

The loss of birds has already impacted Guam’s native forests by altering patterns of seed dispersal (Caves et al. 2013; Rogers et al. 2017; Wandrag et al. 2017) and increasing spider abundance (Rogers et al. 2012). The results presented here, along with those of Rogers et al. (2012), suggest that bird loss has also affected the abundance and community composition of non-native ants and HPIs. While further study is needed to determine the full impacts that ant–HPI associations may have for Guam’s forests, results from other island systems indicate that they will contribute to the decline of native tree species (O’Dowd et al. 2003). Furthermore, the increased densities of ants now present on Guam may hinder efforts to reintroduce birds because of ant disruption of bird nesting, reproductive behaviors, and frugivory (Davis et al. 2008, 2010).

In conclusion, this study provides the first evidence for increased prevalence of honeydew-producing insects and their tending ants in response to landscape-level reduction or loss of birds. These results are consistent with small-scale manipulative experiments that exclude birds and highlight the important role that birds can play as apex predators in terrestrial systems. Understanding how common ecological interactions—including those between ants and HPIs—may change with removal of apex predators such as birds is especially important given the sensitivity of insectivorous birds to habitat loss and fragmentation (Şekercioḡlu et al. 2002) and broader global trends of decreasing avian diversity and abundance (Gaston et al. 2003; Inger et al. 2015).

## Electronic supplementary material

Below is the link to the electronic supplementary material.

Supplementary material 1 (DOCX 1366 kb)
