# Supplementary material for: Landscape-level bird loss increases the prevalence of honeydew-producing insects and non-native ants
Source: Oecologia. 2018 Oct 26;188(4):1263–72. doi: 10.1007/s00442-018-4273-5 (PMC6244808; doi:10.1007/s00442-018-4273-5)
Supplement: Supplementary file 1 — Supplementary material 1 (DOCX 1366 kb) [file 442_2018_4273_MOESM1_ESM.docx]

| **Ant species** | **Guam** | **Rota** | **Saipan** |
| --- | --- | --- | --- |
| ***Anoplolepis gracilipes*** |  |  |  |
| ***Tapinoma melanocephalum*** |  |  |  |
| *Paraparatrechina minutula* |  |  |  |
| *Nylanderia bourbonica* |  |  |  |
| ***Monomorium floricola*** |  |  |  |
| *Odontomachus simillimus* |  |  |  |
| *Crematogaster biroi* |  |  |  |
| *Pheidole oceanica* |  |  |  |
| ***Tetramorium bicarinatum*** |  |  |  |
| *Ochetellus glaber* |  |  |  |
| *Camponotus sp.* |  |  |  |
| ***Technomyrmex albipes*** |  |  |  |
|  | | | |

**Table S1**—Ant species and their presence/absence on each of the three islands sampled. Dark grey cells indicate ant species sampled on the island during our study; light grey cells indicate ant species known to occur on the island but that were not encountered during this study. Species in bold are notable for their widespread distributions and have been recorded as highly invasive in other systems. Specimens with unknown identities (n = 6) are not included here.

| **Model 1 – Response: HPI Presence** | | | | |
| --- | --- | --- | --- | --- |
| **Predictors** | **k** | **AICc** | **ΔAICc** | **Weight** |
| Island + Species + Ant Presence | 17 | 203.8 | 0 | 0.85 |
| Island*Ant Presence + Species | 19 | 207.3 | 3.5 | 0.15 |
| Species*Ant Presence + Island | 28 | 216.4 | 12.6 | 0 |
| Island*Species + Ant Presence | 36 | 222.4 | 18.6 | 0 |
| Island*Species*Ant Presence | 61 | 289.0 | 85.2 | 0 |
| **Model 2 – Response: Ant Presence** | | | | |
| **Predictors** | **k** | **AICc** | **ΔAICc** | **Weight** |
| Island + Species + HPI Presence | 17 | 243.2 | 0 | 0.80 |
| Island*HPI Presence + Species | 19 | 246.1 | 2.8 | 0.20 |
| Species*HPI Presence + Island | 28 | 258.1 | 14.9 | 0 |
| Island*Species + HPI Presence | 36 | 267.2 | 24.0 | 0 |
| Island*Species*HPI Presence | 53 | 309.9 | 66.6 | 0 |
| **Model 3 – Response: Ant Abundance** | | | | |
| **Predictors** | **k** | **AICc** | **ΔAICc** | **Weight** |
| Island + Species + HPI Presence | 17 | 1025.4 | 0 | 0.78 |
| Island*HPI Presence + Species | 19 | 1028.0 | 2.6 | 0.22 |
| Island*Species + HPI Presence | 35 | 1049.2 | 23.8 | 0 |
| Species*HPI Presence + Island | 27 | 1050.9 | 25.6 | 0 |
| Island*Species*HPI Presence | 52 | 1082.2 | 56.8 | 0 |

**Table S2**—Model selection tables for each of the models evaluated in this study. In all cases, model selection favored a model without any interaction terms between fixed effects. All models also included a random intercept for site, and models 1 and 2 included leaf number nested with species as a random slope. K refers to the degrees of freedom for each model; AICc is Akaike’s Information Criterion for each model, corrected for small sample sizes; ΔAICc refers to the change in AICc score between a given model and the best-performing model in that analysis and is used to determine the weight given to each model.


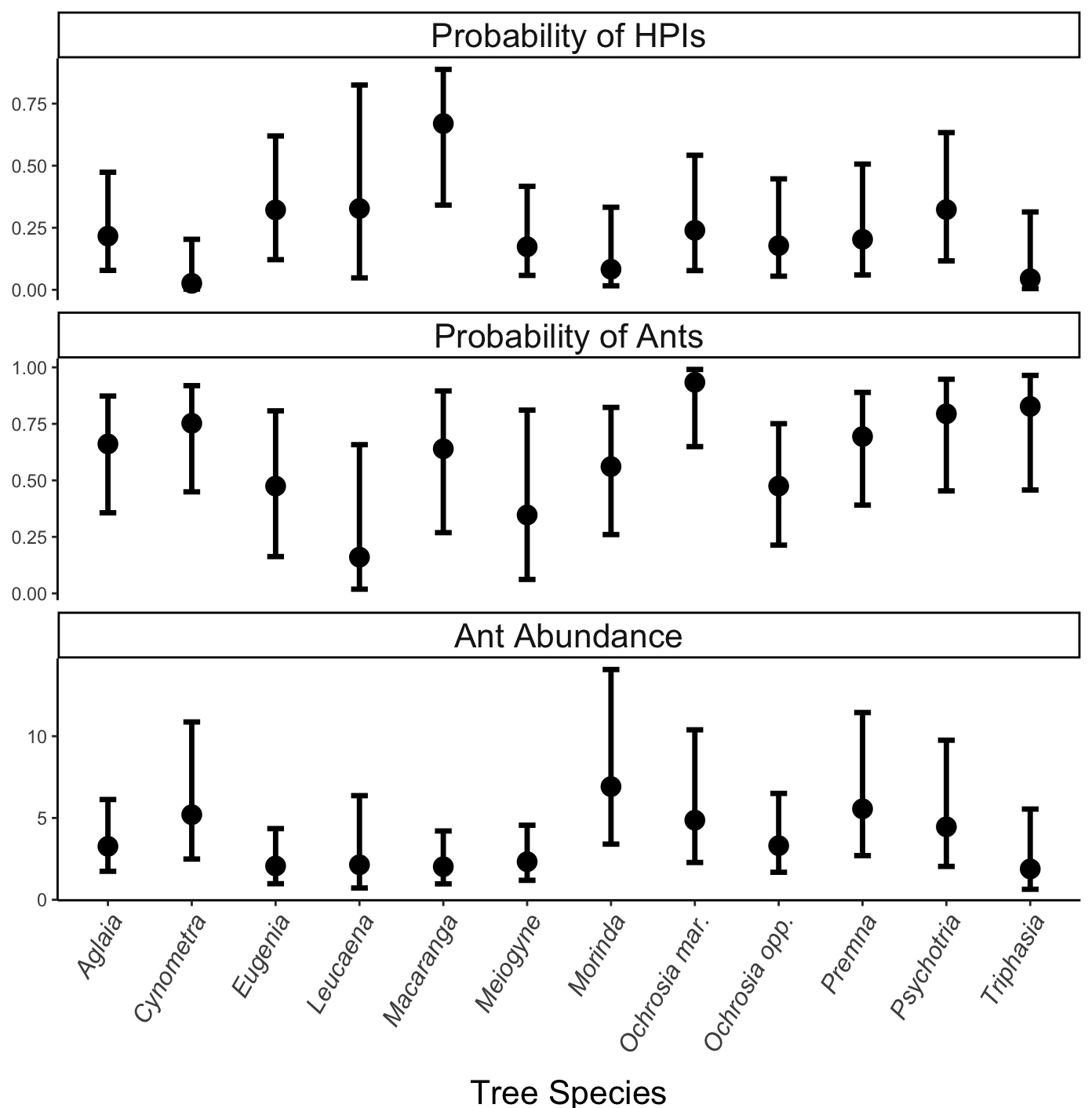


**Figure S1** – Model predictions for probability of encountering of HPIs, probability of encountering ants, and ant abundance for each tree species. Error bars represent 95% confidence intervals. The only significant pairwise difference in any of the above comparisons is between *Macaranga* and *Cynometra* for HPI presence.


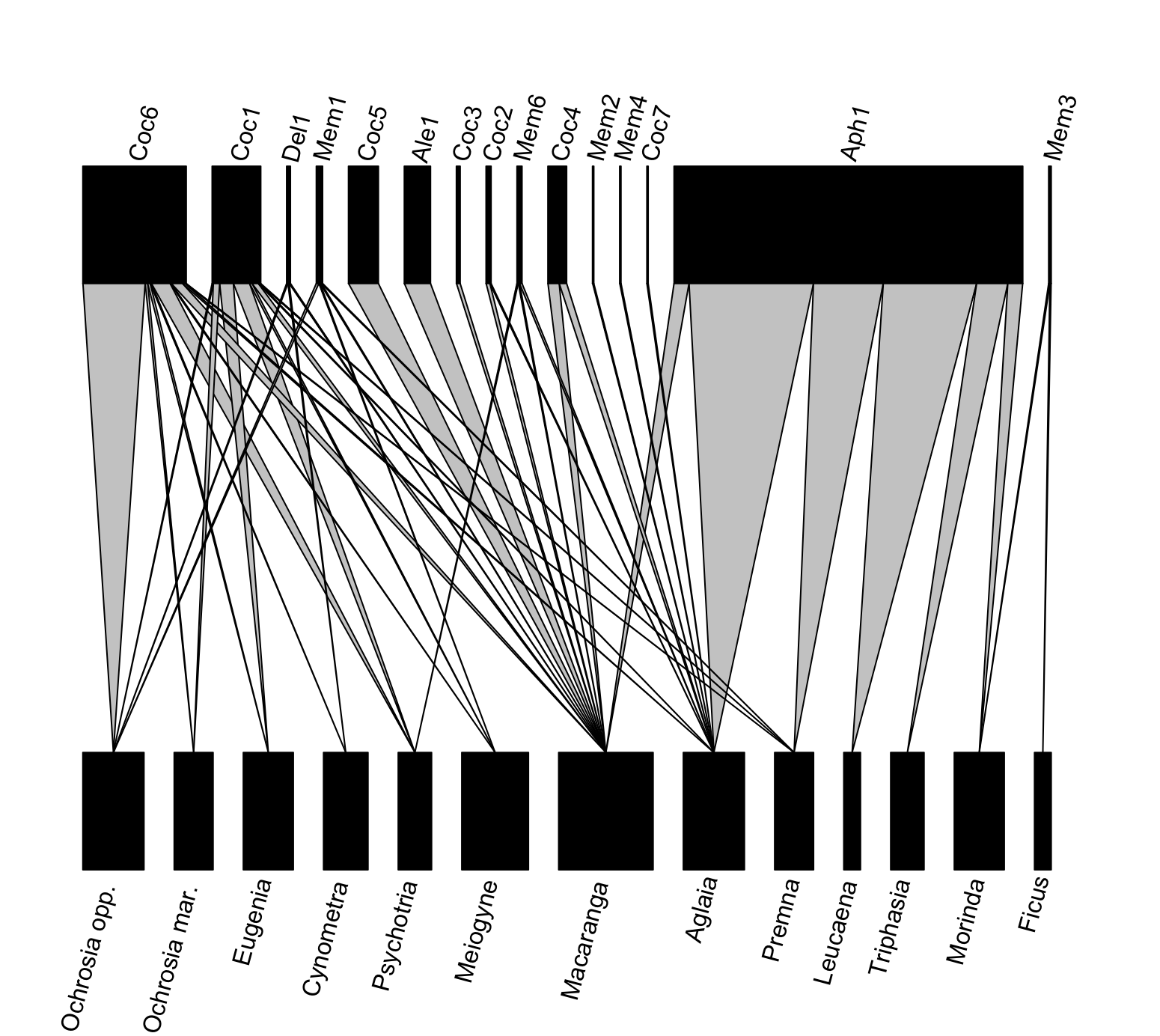


**Figure S2** – Bipartite network showing all pairwise interactions between HPIs and host tree species. Width of bars for HPIs reflect counts of individuals on sampled branches. Abbreviations for HPIs are as follows: Coc = Coccoidea, Del = Delphacidae, Mem = Membracoidea, Ale = Aleyrodidae, Aph = Aphididae.


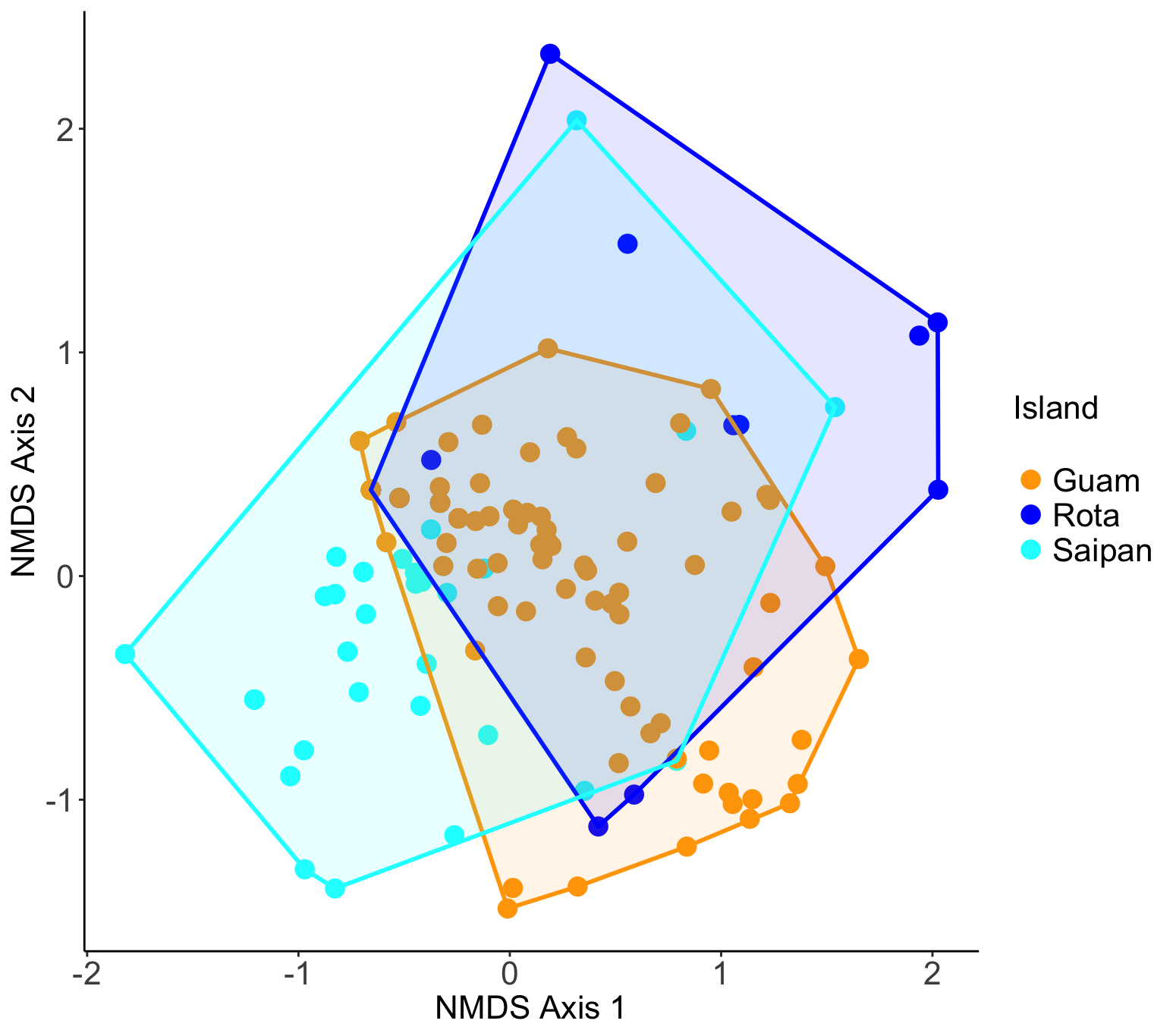


**Figure S3** – NMDS analysis of herbivore communities across islands. Ordination is based on Bray-Curtis distances generated with k = 6 dimensions and a stress value of 0.04. Herbivore communities are driven much less by island (PERMANOVA F_2,138_ = 6.95, R^2^ = 0.06) and more by tree species (F_12_,_138_ = 3.61, R^2^ = 0.20). This is in contrast to ant communities, for which island identity (F_2_,_114_ = 241.6, R^2^ = 0.60) was far more important than tree species (F1_2_,_114_ = 2.62, R^2^ = 0.04).


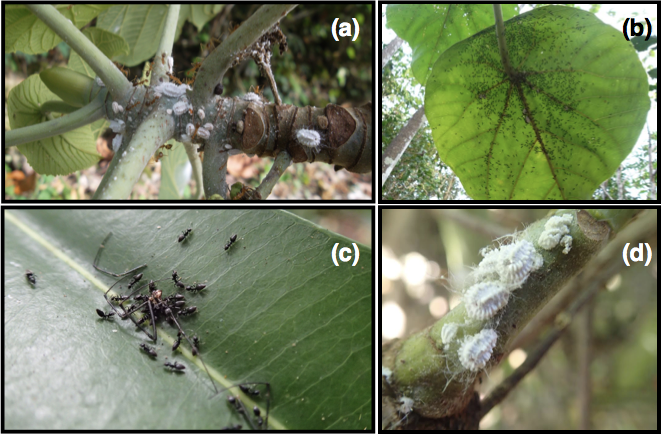


**Figure S4** – (a) Yellow crazy ants (*Anoplolepis* *gracilipes*) tending mealybugs on new growth of *Macaranga thompsonii* from Rota. (b) *Technomyrmex albipes* tending mealybugs on the underside of mature *M. thompsonii* leaves on Guam. Note extremely high ant densities and positioning of mealybugs along the major leaf veins to feed on phloem sap. (c) *Technomyrmex albipes* carrying a dead *Nephila* spider on Guam. (d) Mealybugs feeding on the petiole of *M. thompsonii*.
